# Recombinant adenovirus vector-mediated human *MDA-7* gene transfection suppresses hepatocellular carcinoma growth in a mouse xenograft model^☆^

**DOI:** 10.1016/S1674-8301(12)60007-4

**Published:** 2012-01

**Authors:** Xinting Pan, Liqun Wu, Jingyu Cao, Weidong Guo, Zusen Wang, Bing Han, Weiyu Hu

**Affiliations:** Department of Hepatobiliary Surgery, the Affiliated Hospital of Medical College of Qingdao University, Qingdao, Shandong 266000, China.

**Keywords:** MDA-7, adenovirus, hepatocellular carcinoma, gene therapy, angiogenesis

## Abstract

Hepatocellular carcinoma is one of the most common tumors in the world. The purpose of the present study was to investigate the inhibitory effects of adenoviral transduction of human melanoma differentiation-associated gene-7 (*MDA-7*) gene on hepatocellular carcinoma, so as to provide a theoretical basis for gene therapy of the disease. The human *MDA-7* gene was cloned into replication-defective adenovirus specific to HepG2 cells using recombinant virus technology. RT-PCR and Western blotting assays were used to determine the expression of human *MDA-7* mRNA and MDA-7 protein in HepG2 cells *in vitro*. Induction of apoptosis by overexpression of the human *MDA-7* gene was determined by flow cytometry. *In-vivo* efficacy of adenoviral delivery of the human *MDA-7* gene was assessed in nude mice bearing HepG2 cell lines *in vivo* by determining inhibition of tumor growth, VEGF and CD34 expression, and microvascular density (MVD). The results showed that AdGFP/MDA-7 induced apoptosis of HepG2 cells *in vitro* and significantly inhibited tumor growth *in vivo* (*P* < 0.05). The intratumoral MVD decreased significantly in the treated tumors (*P* < 0.05). We conclude the recombination adenovirus AdGFP/MDA-7 can effectively express biologically active human *MDA-7*, which leads to inhibition of hepatocellular carcinoma growth.

## INTRODUCTION

Hepatocellular carcinoma (HCC) is one of the most common tumors worldwide. Surgical resection is recognized as the most effective method for treatment of HCC. Unfortunately, only a minority of patients currently diagnosed with HCC may benefit from this radical option as most patients are diagnosed in advanced stages[1],[2].

The melanoma differentiation-associated gene-7 (*MDA-7*) is a novel tumor suppressor gene belonging to the IL-10 cytokine superfamily. It was first identified in 1995 by subtraction hybridization of a cDNA library derived from a human HO-1 melanoma cell line treated with interferon (IFN)-β and protein kinase C activator, mezerin[3]. Overexpression of MDA-7 has been shown to induce tumor cell apoptosis *in vitro* in a wide variety of cancer cells including melanoma, breast and colorectal cancer[4]-[6]. Furthermore, MDA-7-induced apoptosis is tumor selective with minimal cytotoxicity observed in normal human cells. Adenovirus can be used to mediate gene transfer in a wide range of cell types and high level transgene expression can be obtained at high titers[7].

In this study, we constructed a recombinant adenovirus expressing green fluorescent protein (GFP) and tumor suppressor MDA-7 (AdvGFP/MDA-7). We observed that AdvGFP/MDA-7 down-regulated vessel expression of vascular endothelial growth factor (VEGF) and CD34, reduced vessel density in tumors, and inhibited tumor growth in a nude mouse tumor model.

## MATERIALS AND METHODS

### Cell line, reagent and mice

Human embryonic kidney cell line (QBI-293A) was cultured in Dulbecco's Modified Eagle's Medium (DMEM; Sigma, China) supplemented with 10% fetal calf serum. Human pancreatic carcinoma cell line HepG2 was maintained in RP1640 medium. Biotin-conjugated anti-CD34, VEGF antibodies and anti-mouse IgG antibody were obtained from Santa Cruz Biotechnology (Shanghai, China). BALB/c nude mice were obtained from Shanghai Laboratory Animal Center, Chinese Academy of Sciences (Shanghai, China) and maintained according to the guidelines for animal experimentation of the Institutional Animal Care and Use Committee at Qingdao University. The study protocol was approved by the local institutional review board.

### Construction of recombinant adenoviral vectors

A pair of primers was designed to clone the human MDA-7 gene directly from RNA of IFN-γ-treated human peripheral blood mononuclear cells by using reverse transcription-polymerase chain reaction (RT-PCR). The sense primer sequence was: 5′-ATGGATATCATGCAGGGCCAAGAATTCCACTT-3′, and the antisense primer sequence 5′-GCACTCGAGTCAGAGCTTGTAGAATTTC-3′. The cloned *MDA-7* cDNA fragment was ligated into pAd-Track-GFP vector expressing GFP to form pAdTrack-GFP/MDA-7 expressing both GFP and human MDA-7. The resultant pAdGFP/MDA-7 and pAdGFP plasmids were purified from transfected BJ5183 cells, and then linearized by *Pac* I digestion. The resulting digest was transfected into human embryonic kidney 293 cells by Lipofectamine, leading to formation of the recombinant adenoviruses AdvGFP and AdvGFP/MDA-7. The AdvGFP/MDA-7 vectors were amplified in QBI-293A cells, and purified by cesium chloride ultracentrifugation, and stored at -80°C.

### Transfection of HepG2 cells with AdvGFP/MDA-7

Exponentially growing human HCC cell line HepG2 was seeded onto a 6-well plate at 1×10^5^ cells per well and incubated for 48 h. The cells were allowed to grow till 80% confluence and the medium was removed. The mixtures were incubated with viral solutions for 12 h. Transfections were terminated by replacing viral solutions with fresh medium. Two days after culturing, fluorescence microscopy was used to observe the expression of GFP in infected HepG2 cells.

### *MDA-7* gene expression in HepG2 cells

Two weeks after transfection, total RNA was extracted from AdvGFP/MDA-7-transfected, AdvGFP-transfected and non-transfected HepG2 cells, respectively. Expression of *MDA-7* mRNA was detected by RT-PCR, and *β-actin* served as internal control. The protocol of RT-PCR was as follows: denaturation at 93°C for 2 min; 30 cycles of denaturation at 94°C for 55 sec, annealing at 55°C for 55 sec and extension at 72°C for 50 sec; final extension at 72°C for 10 min. The reaction products were electrophoresed on 15% agarose gel and photographed. The calculated data represented the expression of *MDA-7* mRNA in each group.

### Western blotting analysis

HepG2 cells were cultured, infected and harvested. Total cell lysates were resolved by SDS-PAGE and transferred to a nitrocellulose membrane. The membrane was blocked in PBS containing 3% (W/V) nonfat dry milk at room temperature for 2 h. The membrane was incubated with primary anti-MDA-7 (1:2,000) antibody in blocking solution at room temperature for 2 h. All membranes were then washed and incubated for 1 h at room temperature, and incubated overnight with alkaline phosphatase-conjugated secondary antibody (1:10,000, Sigma, Oakville, Ontario, Canada).

### Detection of apoptotic cell by flow cytometry

HepG2 cells (5×10^5^) reaching 70% confluence were infected with AdvGFP and AdvGFP/MDA-7. Two d later, cells were harvested after digestion by 0.25% trypsin, washed with PBS, and fixed in 70% ethanol overnight. Cells were resuspended in 1 mL of PBS containing 1 mg/mL RNaseA and 0.5 mg/mL propidium iodine (Sigma, Oakville, Ontario, Canada). After 30 min of incubation, cells were analyzed by flow cytometry.

### *In vivo* experiment

Male BALB/c nude mice (10 mice each group) were inoculated subcutaneously in the flank with 1×10^7^ HepG2 cells. Two weeks later, when the tumors were 0.2-0.3 cm in size, the mice were injected intratumorally with PBS or AdvGFP or AdvGFP/MDA-7 (1×10^7^ pfu/50 µL) each day for 5 d and tumor growth was monitored daily. Tumor size was calculated using the following formula: *V* = *ab*^2^/2, where *V* represents the tumor size, *a* is the larger and *b* is the smaller of the two dimensions. For immunohistochemistry, VEGF and CD34 were measured with immunohistochemical kits. Brown staining in or around the nucleus was taken as positive immunoreactivity for VEGF and CD34. One lumen of blood vessels was assessed as a new blood capillary.

### Statistical analysis

All data were expressed as mean±SD, and all statistical analyses were performed using the statistical software SPSS 13.0. Statistically significant differences were tested by Student's *t*-test and one-way analysis of variance (ANOVA) as appropriate. A *P* value < 0.05 was considered significant.

## RESULTS

### Transfection of HepG2 cells with AdvGFP/MDA-7

**Fig. 1:**
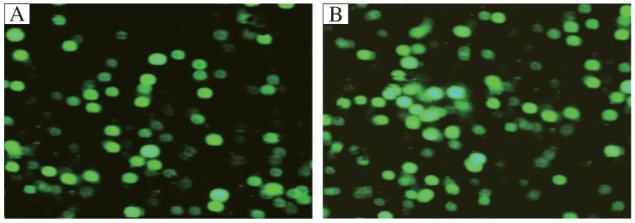
Expression of hMDA-7 in HepG2 cells infected with AdvGFP/MDA-7. HepG2 cells were imaged by fluorescence microscopy following infection with AdvGFP/MDA-7 and AdvGFP. A: AdvGFP. B: AdvGFP/MDA-7.

The recombinant adenoviral vector was validated by restriction enzyme digestion and DNA sequencing. The results demonstrated that the *MDA-7* cDNA sequence was consistent with that in the GenBank, which indicated that the recombinant adenoviral vector AdvGFP/MDA-7 was constructed successfully. To determine the optimal multiplicity of infection (MOI) for a maximal transgene expression, HepG2 cells were infected with AdvGFP/MDA-7 at various MOIs and examined by fluorescence microscopy. At MOI of 10 and higher, more than 95% of the HepG2 cells transfected with AdvGFP/MDA-7 were GFP positive (***Fig. 1***). Therefore, an MOI of 10 was selected as the optimal dose for transfection of HepG2 cells.

### MDA-7 expression in HepG2 cells

**Fig. 2:**
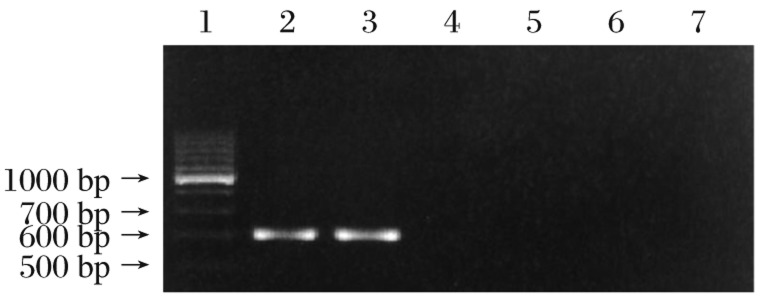
Expression of MDA-7 mRNA in HepG2 cells determined by RT-PCR. *MDA-7* mRNA was stably expressed in HepG2 cells infected with AdvGFP/MDA-7, while no expression was detected in cells infected with AdvGFP or PBS. Lane 1: molecular marker; Lane 2 and 3: AdvGFP/MDA-7 infected cells; Lane 4 and 5: AdvGFP-transfected cells; Lane 6 and 7: uninfected HepG2 cells. The target band is 621 bp.

**Fig. 3:**
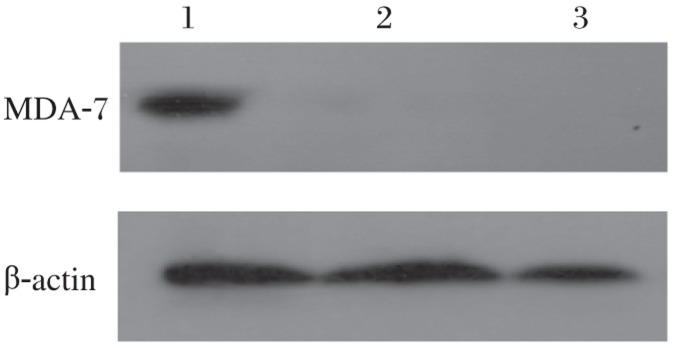
Expression of *MDA-7* mRNA in HepG2 cells detected by Western blotting assay. Human MDA-7 was positive only in AdvGFP/MDA-7 transfected HepG2 cells. Lane 1: AdvGFP/MDA-7-transfected cells; Lane 2: AdvGFP-transfected cells; Lane 3: non-transfected cells.

RT-PCR analysis demonstrated that *MDA-7* mRNA was stably expressed in HepG2 cells infected with AdvGFP/MDA-7 (***Fig. 2***), while no expression of *MDA-7* mRNA was detected in HepG2 cells infected with AdvGFP or PBS. The expression of β-actin was not observably altered with any of the transfections. To investigate the expression of *MDA-7* in AdvGFP/MDA-7-transfected HepG2 cells, we examined the expression of human *MDA-7* examined by Western blot analysis. We found that human *MDA-7* was only expressed in AdvGFP/MDA-7-transfected HepG2 cells (***Fig. 3***), demonstrating AdvGFP/MDA-7 mediated effective infection and specific expression of the human *MDA-7* gene in HepG2 cells.

### MDA-7 expression induces apoptosis of HepG2 cells

**Fig. 4:**
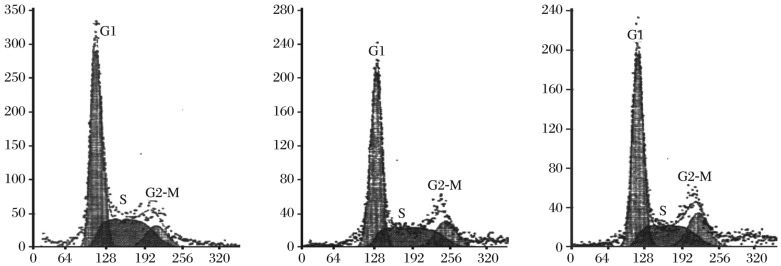
Cell cycle distribution of HepG2 cells by flow cytometry. AdvGFP/MDA-7 induced alteration of cell cycle distribution with a reduction in the number of cells in the S-phase and activation of G2/M-phase arrest. A: PBS group; B: AdvGFP group; C: AdvGFP/MDA-7 group.

As shown in ***Fig. 4***, AdVGFP/MDA-7 transfection induced alterations in cell cycle distribution *in vitro* with a reduction in the number of cells in the S-phase and caused a G2/M-phase arrest. However, our flow cytometric studies revealed no alteration of cell cycle in HepG2 cells transfected with AdVGFP or PBS.

### AdvGFP/MDA-7 suppresses tumor growth *in vivo*

**Fig. 5:**
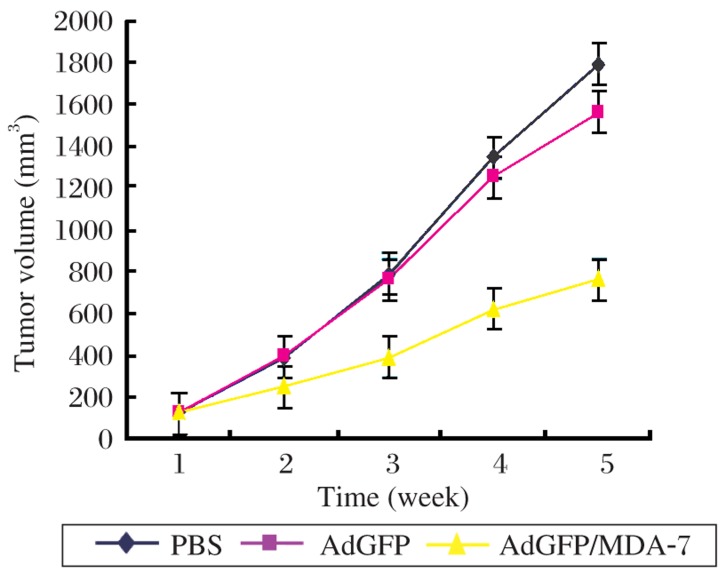
Inhibition of growth of tumors infected with AdvGFP/MDA-7, AdvGFP and PBS in nude mice. Compared to AdvGFP and PBS groups, *P* < 0.05.

Treatment of nude mice bearing xenografts of HepG2 tumor cells infected with AdvGFP/MDA-7 induced a significant reduction in tumor growth compared with those treated with AdvGFP [AdvGFP/MDA-7, (763.23±16.11) mm^3^ *vs* AdvGFP (1563.43±19.26) mm^3^ or PBS (1 795.62±19.70) mm^3^, *P* < 0.05 in both]. The results indicated that AdvGFP/MDA-7 could effectively suppress tumor growth *in vivo* (***Fig. 5***).

### AdvGFP/MDA-7 down-regulates the expression of VEGF and CD34

**Fig. 6:**
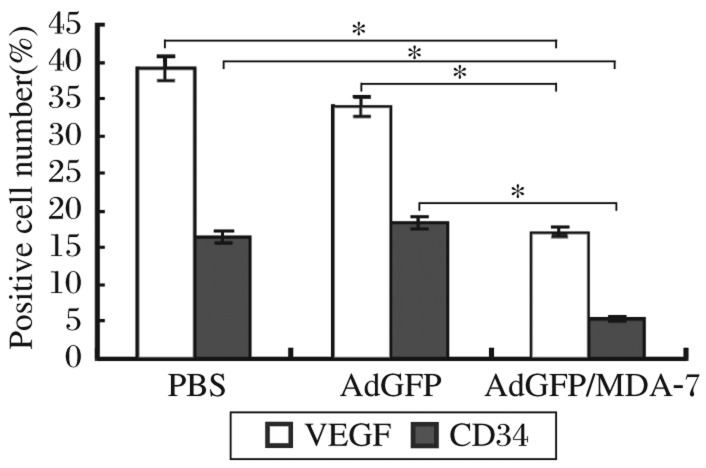
Immunohistochemical staining of VEGF and CD34 in tumor tissues infected with AdvGFP/MDA-7, AdvGFP and PBS. AdvGFP/MDA-7 treatment significantly down-regulated the expression of VEGF and CD34 in tumor cells infected with AdvGFP/MDA-7, AdvGFP and PBS, *P < 0.05.

As a possible mechanistic explanation for AdvGFP/MDA-7-induced reduction in tumor growth, the ability of AdvGFP/MDA-7 to alter the expression of VEGF and CD34 in tumors was assessed. AdvGFP/MDA-7 treatment significantly down-regulated the expression of VEGF and CD34 in tumors infected with AdvGFP/MDA-7 compared with those infected with AdvGFP and PBS (***Fig. 6***, *P* < 0.05), suggesting that down-regulation of VEGF and CD34 expression was a possible mechanism whereby AdvGFP/MDA-7 inhibited tumor growth *in vivo*. In addition, the intratumoral microvascular density (MVD) decreased significantly in tumors treated with AdvGFP/MDA-7 (5.5±0.8) compared with those treated with AdvGFP (18.3±1.2) or PBS (16.5±1.5) (*P* < 0.05 in both).

## DISCUSSION

As one of the world's most common malignancies, HCC is the third most common cause of cancer-related deaths, and in recent years the incidence of the disease has been increasing. Difficulty in early diagnosis of HCC results in poor prognosis. Non-surgical therapy, such as radiotherapy, chemotherapy and gene therapy, plays important roles in the treatment of patients with HCC[8]. However, the effect of adenovirus-mediated human MDA-7 on the disease remains unknown till now. MDA-7 was discovered in 1995, and identified by subtractive hybridization of melanoma cells following treatment with IFN-β and mezerein, which caused terminal differentiation and growth arrest. MDA-7 is a secreted protein with cytokine-like properties and belongs to the IL-10 cytokine family, which includes IL-10, IL-19, IL-20, IL-22 and IL-26.

AdVMDA-7-mediated cancer gene therapy has been demonstrated to be cytotoxic *in vitro* to various tumor cells. The *in-vivo* tumor suppression effect of AdVMDA-7 has been proved in various animal tumor models. This anti-tumor effect of MDA-7 is independent of classical tumor suppressor genes, such as *p53*, *Rb* and *p16*[9]-[11]. In this study, we constructed an adenovirus vector containing both GFP and MDA-7. Our findings demonstrated that tumor cells infected with AdvGFP/MDA-7 at an MOI of 10 resulted in GFP expression in more than 98% of the cells, indicating that this construct exhibited high infectivity. Expression of human *MDA-7* was observed in AdvGFP/MDA-7-transfected HepG2 cells, demonstrating the specific expression of the human *MDA-7* gene in HepG2 cells.

The decision of cells to differentiate is commonly made in the G1 phase of the cell cycle[20]. The regulation of cells entering from the G1 phase of the cell cycle into the S phase is particularly important, as normal cells must pass through a checkpoint in late G1 to progress to the S phase. In this study, AdvGFP/MDA-7 was demonstrated to induce apoptosis of HepG2 cells *in vitro*. Treatment of HepG2 cells with AdvGFP/MDA-7, but not AdvGFP or PBS, resulted in a significant reduction in the number of cells in the S phase concomitant with an increase in the number of cells in the G2/M phase, which was consistent with findings of a previous study[13].

Tumor growth relies on angiogenesis, the formation of new blood vessels, to receive an adequate supply of oxygen and nutrients[14]. VEGF is an important regulator in the growth of many solid tumors conferring survival advantage by inducing vascular formation via stimulation of endothelial cells[15]. Angiogenesis in HCC is based on the same fundamental principles of activation, proliferation and migration of endothelial cells. Overexpression of angiogenic genes such as *VEGF* and *CD34* has been shown to be associated with enhanced tumorigenicity and tumor metastatic potential[16],[17]. CD34 is a cell surface marker of progenitor cells, and is frequently used as an indicator of newly formed vessels[18]. In this study, we demonstrated that *in-vivo* expression of angiogenesis-associated molecules CD34 and VEGF was significantly down-regulated in tumors treated with AdvGFP/MDA-7 compared with those in AdvGFP-treated tumors. Moreover, AdvGFP/MDA-7 treatment resulted in reduced MVD within tumors. Our findings indicate that down-regulation of VEGF and CD34 expression is a possible mechanism underlying AdvGFP/MDA-7-mediated inhibition of HCC growth *in vivo*.

In summary, AdvGFP/MDA-7 can replicate in HepG2 cells and induce cellular apoptosis. It also down-regulates expression of angigenic genes *VEGF* and *CD34*, resulting in reduced tumor vessel formation. In an animal tumor model, AdvGFP/MDA-7 gene therapy significantly inhibited HCC growth and prolonged the survival of tumor-bearing nude mice. It is therefore concluded that adenovirus-mediated human MDA-7 gene therapy may serve as a novel therapeutic approach for HCC.

## References

[1] Kim HO, Kim SK, Son BH, Yoo CH, Hong HP, Cho YK (2009). Intraoperative radiofrequency ablation with or without tumorectomy for hepatocellular carcinoma in locations difficult for a percutaneous approach. Hepatobiliary Pancreat Dis Int.

[2] Sandonato L, Cipolla C, Fulfaro F, Lo Re G, Latteri F, Terranova A (2009). Minor hepatic resection using heat coagulative necrosis. Am Surg.

[3] Gupta P, Walter MR, Su ZZ, Lebedeva IV, Emdad L, Randolph A (2006). BiP/GRP78 is an intracellular target for MDA-7/IL-24 induction of cancer-specific apoptosis. Cancer Res.

[4] Zheng M, Bocangel D, Doneske B, Mhashilkar A, Ramesh R, Hunt KK (2007). Human interleukin 24(MDA-7/IL-24)protein kills breast cancer cells via the IL-24 receptor and is antagonized by IL-24. Cancer Immunol Immunother.

[5] Lebedeva IV, Su ZZ, Chang Y, Kitada S, Reed JC, Fisher PB (2002). The cancer growth suppressing gene mda-7 induces apoptosis selectively in human melanoma cells. Oncogene.

[6] Su Z, Lebedeva IV, Gopalkrishnan RV, Goldstein NI, Stein CA, Reed JC (2001). A combinatorial approach for selectively inducing programmed cell death in human pancreatic cancer cells. Proc Natl Acad Sci USA.

[7] Zhou LF, Yin KS, Zhu ZL, Zhu Y, Yao X, Mao H (2005). Adenovirus-mediated overexpression of novel mutated IκBα inhibits nuclear factor κB activation in endothelial cells. Chin Med J(Engl).

[8] McIntosh A, Hagspiel KD, Al-Osaimi AM, Northup P, Caldwell S, Berg C (2009). Accelerated treatment using intensity-modulated radiation therapy plus concurrent capecitabine for unresectable hepatocellular carcinoma. Cancer.

[9] Wei L, Wang Z, Cui T, Yi F, Bu Y, Ding S (2008). Proteomic analysis of cervical cancer cells treated with adenovirus-mediated MDA-7. Cancer Biol Ther.

[10] Yang J, Zhang W, Liu K, Jing S, Guo G, Luo P (2007). Expression, purification, and characterization of recombinant human interleukin 24 in Escherichia coli. Protein Expr Purif.

[11] Gopalan B, Shanker M, Chada S, Ramesh R (2007). MDA-7/IL-24 suppresses human ovarian carcinoma growth *in vitro* and *in vivo*. Mol Cancer.

[12] Pardee AB (1989). G1 events and regulation of cell proliferation. Science.

[13] Gupta P, Su ZZ, Lebedeva IV, Sarkar D, Sauane M, Emdad L (2006). mda-7/IL-24: multifunctional cancer-specific apoptosis-inducing cytokine. Pharmacol Ther.

[14] Semenza GL (2003). Angiogenesis in ischemic and neoplastic disorders. Annu Rev Med.

[15] Zhou Z, Zhou RR, Guan H, Bucana CD, Kleinerman ES (2003). E1A gene therapy inhibits angiogenesis in a Ewing's sarcoma animal model. Mol Cancer Ther.

[16] Gasparini G (2000). Prognostic value of vascular endothelial growth factor in breast cancer. Oncologist.

[17] Vieira SC, Silva BB, Pinto GA, Vassallo J, Moraes NG, Santana JO (2005). CD34 as a marker for evaluating angiogenesis in cervical cancer. Pathol Res Pract.

[18] Perryn ED, Czirók A, Little CD (2008). Vascular sprout formation entails tissue deformations and VE-cadherin-dependent cell-autonomous motility. Dev Biol.

